# Hypocalcemia-Induced Reversible Psychosis

**DOI:** 10.7759/cureus.20874

**Published:** 2022-01-02

**Authors:** Andrea Hall, Talha Bin Farooq, Renato Alcaraz Jr.

**Affiliations:** 1 Internal Medicine, Carle Illinois College of Medicine, Urbana, USA; 2 Internal Medicine, Carle Foundation Hospital, Urbana, USA

**Keywords:** reversible, delusions, hypoparathyroidism, metabolic psychosis, s: hypocalcemia

## Abstract

Hypocalcemia can manifest as a variety of presentations, ranging from neuromuscular irritability to seizures, and psychiatric manifestations such as emotional instability, anxiety, and depression. Here, we present a unique case of hypocalcemia-induced acute psychosis.

A 24-year-old woman presented to the emergency department (ED) with confusion and agitation for four to five days. The patient was noted by the family to have decreased oral intake and sleep. Auditory and visual hallucinations prompted the family to bring the patient to the ED. The patient was mildly tachycardic. Initially, the patient was agitated, impulsive, and aggressive, exhibiting psychotic features including visual hallucinations, paranoid delusions, thought broadcasting, tangential and perseverative thought processes, and erotomanic delusions. She had mild leukocytosis and elevated procalcitonin on admission. A thorough workup ruled out infectious/inflammatory processes. Cerebrospinal fluid was negative for acute meningitis/encephalitis, autoimmune encephalitis antibodies, and paraneoplastic etiology. Thyroid-stimulating hormone was normal and thyroid antibodies were negative. The CT brain and MRI brain were unremarkable. The patient was severely hypocalcemic (6.7) with low parathyroid hormone (<6) on admission.

To note, the patient has multiple endocrine neoplasia, type 2B (MEN2B). She underwent total thyroidectomy five months prior for metastatic medullary thyroid carcinoma complicated by postsurgical hypoparathyroidism. The patient had been non-compliant with calcium and calcitriol supplementation postoperatively.

The patient was started on IV calcium gluconate and transitioned to calcitriol with calcium level improvement over the next three days. She experienced marked improvement, with the resolution of her psychosis. The patient’s subacute onset psychosis with no personal or family psychiatric history and a rapid response to calcium correction supports hypocalcemia etiology.

This case illustrates new-onset acute psychosis in a patient with calcium regulation imbalance. The development of hypocalcemia secondary to thyroidectomy with postsurgical hypoparathyroidism and calcium supplement non-compliance precipitated psychosis. A few similar cases have been reported, and here, we note that treatment of hypocalcemia promptly resolves symptoms. As per our review, this will be the first case of neuropsychiatric symptoms without associated cortical calcifications seen on imaging. It is important to recognize hypocalcemia as a rare cause of psychosis so as to not mistakenly categorize such presentations as primary psychotic disorders and miss a medically treatable illness.

## Introduction

Hypocalcemia may present as an asymptomatic laboratory finding or possibly as a life-threatening illness. Patients most often present with neuromuscular irritability, including muscle cramps, paresthesia, tetany, and seizures. Neuropsychiatric symptoms have usually been associated with chronic hypocalcemia in patients with hypoparathyroidism associated with calcification of the basal ganglia [1,2].

We present a case of psychosis induced by hypocalcemia in a young patient with hypoparathyroidism with no other signs or symptoms of hypocalcemia.

## Case presentation

A 24-year-old female with past medical history significant for medullary thyroid carcinoma (multiple endocrine neoplasia, type 2B, MEN2B) status post thyroidectomy, post-surgical hypoparathyroidism, and pheochromocytoma status post complete left and partial right adrenalectomy, presented with confusion, altered mentation, and poor oral intake over the last four to five days. The patient was noted by her husband as having these symptoms. She had not been sleeping well for the last two weeks, and for the last four days had not slept at all. She was unable to recognize her in-laws. She typically provides the majority of the care for her seven-month-old, but had not been doing this for the last three days. The patient had no history of any psychiatric illness, including postpartum depression.

The patient was also experiencing auditory and visual hallucinations along with a mild headache. She would sometimes hear drumming sounds and report seeing yellow and green colors in the room. She would reply to most questions by either saying "no" or "you." She was also making hand gestures toward members of the care team.

Upon presentation, the patient was alert but completely disoriented. She was not following commands. Her blood pressure was 106/65 mmHg, her respiratory rate was 18, and she was afebrile and slightly tachycardic at 105 bpm. Urinalysis showed no signs of infection. An EKG showed sinus tachycardia with a normal QTc interval. Chest X-rays did not show any consolidation or fluid collection. A CT scan of the brain did not show any abnormalities. Laboratory findings are given in Table 1.

**Table 1:** Laboratory findings

	Patient’s value	Reference range
White blood cells	11,200/μL	4000–11,000/μL
Calcium	6.7 mg/dL	8.4–10.1 mg/dL
Creatinine	0.59 mg/dL	0.52–1.04 mg/dL
Sodium	138 mmol/L	137–145 mmol/L
Potassium	4.2 mmol/L	3.5–5.1 mmol/L
Phosphorus	3.7 mg/dL	2.5–5.0 mg/dL
Albumin	3.8 g/dL	3.4–5.0 g/dL
Magnesium	2.0 mg/dL	1.6–2.6 mg/dL
Parathyroid hormone	<6 pg/mL	14–72 pg/mL
Procalcitonin	55 ng/mL	<0.5 ng/mL
Thyroid-stimulating hormone	0.364 mcIU/mL	0.358–3.740 mcIU/mL
Free T4 levels	1.42 ng/dL	0.76–1.46 ng/dL
Free plasma metanephrine	<0.20 nmol/L	<0.50 nmol/L
25-hydroxy vitamin D	38.7 ng/mL	30–100 ng/mL

Due to leukocytosis, significant procalcitonin elevation and headache, it was imperative that meningitis and encephalitis be ruled out. Neurology recommended lumbar puncture (LP) under general anesthesia and an MRI to rule out any metastasis. An LP was performed and did not show any signs of infection. While awaiting results, IV vancomycin, ceftriaxone, and acyclovir have been provided empirically. Meanwhile, the patient continued to be increasingly physically aggressive during interactions with team members. She continued to remain completely disoriented. She also made sexual comments towards team members. She refused to take oral calcitriol and calcium supplementation; therefore, she was treated with intravenous calcium gluconate.

After CSF infection was ruled out, antimicrobials were discontinued. By day three, the patient started to improve, and she was observed to be calmer and more oriented. Psychiatry started the patient on aripiprazole 5 mg/day on day 3, due to the presentation of psychosis and differentials including new-onset psychiatric disorder, but discontinued it after one day. Psychotic symptoms resolved. The patient was discharged from the hospital in stable condition when she was back to her baseline mental status.

On follow-up with her endocrinologist two months after discharge, she was stable with no further episodes of psychosis. She did not complain of any symptoms at this time and was caring for her daughter and seeking management options for her MEN 2B syndrome.

## Discussion

Hypocalcemia is very commonly seen in healthcare settings. It may be asymptomatic, but when it is symptomatic, it is usually associated with neuromuscular excitability. Some common manifestations include paresthesia, tetany, muscle cramps, seizures, dystonia, confusion, abdominal pain, and a prolonged QT interval on EKG [2]. Hypocalcemia has also been associated with some psychiatric manifestations, including depression, anxiety, and delirium. Acute psychosis is very rare in cases of hypocalcemia [3]. There have been only a few cases reported regarding hypocalcemia associated with severe psychosis. Hypoparathyroidism can lead to elevated calcium-phosphorus products and can lead to calcium deposition in soft tissues, including the brain. Some case reports describe chronic hypocalcemia leading to progressively worsening hallucinations associated with basal ganglia calcification. There have also been some cases associated with frontal cortical calcifications [1,4-7].

Some of the most common causes of hypocalcemia are vitamin D deficiency, hypomagnesemia, alcoholism, acute pancreatitis, and multiple blood transfusions. One of the most important causes of hypocalcemia is postsurgical hypoparathyroidism, most commonly as a complication of thyroidectomy. There has also been an association of hypomagnesemia contributing to resistance to parathyroid hormone action and leading to persistence of symptoms even after serum calcium levels have been corrected [3,5].

It is important to rule out other more common causes of acute delirium and psychosis while keeping a broad differential, especially in a patient with hypoparathyroidism. Hypocalcemia should still be included in the differential even if the patient does not have any other signs or symptoms associated with hypocalcemia. In our patient, symptomatic improvement directly correlated with serum calcium levels, with the patient returning to her baseline mental status when serum calcium levels were around 8.5 mg/dl. Non-compliance with vitamin D and calcium supplements can be one of the major risk factors in such patients. It is important to recognize that even in the absence of cortical calcifications, improvement in calcium levels can rapidly improve a patient’s symptoms.

## Conclusions

Neuropsychiatric symptoms have been associated with hypocalcemia. In most cases, they have been associated with basal ganglia or frontal cortical calcifications, even in cases of acute presentations. As per our review of the literature, this will be the first case reported with acute psychotic symptoms presenting in a patient with hypocalcemia with no cortical calcifications. Therefore, it is important to recognize hypocalcemia as a cause of acute psychosis that can be corrected and lead to rapid improvement of a patient’s symptoms.
